# Antibacterial Attributes of Apigenin, Isolated from *Portulaca oleracea L.*

**DOI:** 10.1155/2014/175851

**Published:** 2014-05-13

**Authors:** Hanumantappa B. Nayaka, Ramesh L. Londonkar, Madire K. Umesh, Asha Tukappa

**Affiliations:** Department of Post Graduate Studies and Research in Biotechnology, Gulbarga University, Gulbarga, Karnataka 585106, India

## Abstract

The flavonoid apigenin was isolated from aerial part of *P. oleracea L*. The dried sample of plant was powdered and subjected to soxhlet extractor by adding 80 mL of ethanol : water (70 : 30). The extract was centrifuged at 11000 rpm for 30 min; supernatant was taken for further use. The fraction was concentrated and subjected to PTLC. The *R*_*f*_ value of isolated apigenin was calculated (0.82). Purified material was also subjected to its IR spectra, LC-MS, NMR, and HPLC for structural elucidation. The apigenin so-obtained was subjected to antibacterial activity on five pathogenic bacterial strains like *Pseudomonas aeruginosa, Salmonella typhimurium, Proteus mirabilis, Klebsiella pneumoniae* and *Enterobacter aerogenes*; among all the bacterial strains, *Salmonella typhimurium *(17.36 ± 0.18) and *Proteus mirabilis *(19.12 ± 0.01) have shown maximum diameter of inhibition zone for flavonoid and remaining bacterial strains have shown moderate diameter of inhibition zone when compared with control values 14.56 ± 0.21 and 11.68 ± 0.13, respectively. The minimum inhibitory concentration (MIC) of the flavonoid isolated from *P. oleracea L.* was tested at the concentration ranging from undiluted sample to 10 mg per mL of concentration. The minimum inhibition concentration (MIC) for the flavonoid for all tested bacterial strains was found to be >4 mg per mL. Hence, the apigenin has antibacterial property and can be used to develop antibacterial drugs.

## 1. Introduction

Plants have potent biochemical components which are phytomedicine. Plant based natural constituents can be derived from any part of the plant like bark, leaves, flowers, roots, fruits, seeds, and so forth, and used as medicine by the man since the time immemorial [1]. The beneficial medicinal effects of plant materials are typically the result of combinations of secondary products present in the plant. The medicinal values of plants are unique to particular plant species or groups and are consistent with this concept as the combination of secondary products in a particular plant is taxonomically distinct [2]. Flavonoids are group of about 4000 naturally occurring polyphenol compounds, found universally in all the plants [3]. These are primarily recognized as the pigments responsible for the colors of leaves, especially in autumn. Flavonoids are widely distributed in fruits, vegetables, nuts, seeds, herbs, spices, stems, and flowers as well as tea and red wine. They are usually subdivided according to their substituents present in flavanols (kaempferol, quercetin), anthocyanins, flavones, flavonones, and chalcones. These flavonoids display a remarkable array of biochemical and pharmacological actions, namely, anti-inflammatory, antioxidant, antiallergic, hepatoprotective, antithrombotic, antiviral, and anticarcinogenic activities [4]. Phytocompounds appear to play a vital role in defense mechanism against pathogens and predators and contribute to physiological functions of plant such as seed maturation and dormancy [5]. They are synthesized from phenyl propanoid and acetate derived precursors. Flavonoids are important for human beings due to their antioxidative and radical scavenging effects as well as their potential as estrogenic and anticancer activities [6]. In recent years, antibiotic resistance has become a global concern and this problem is more important in developing country because the infectious diseases are still an important cause of morbidity and mortality among humans. Plants rudely synthesize substances for their defense against insects, herbivores, and microorganisms [7]. Nowadays, multiple drug resistance is developed due to the indiscriminate use of drugs which are commonly used in the treatment of infectious diseases [8]. In addition to this problem, antibiotics are sometimes associated with adverse effect on the host including hypersensitivity, immune suppression, and allergic reaction [9]. Because of side effect and resistance that the pathogenic microorganism developed against antibiotics, recently much attention has been paid to extraction of biologically active compounds from plants. Plant based antimicrobials represent a vast updated source of medicine. Antibacterials of plant origin have enormous therapeutic potential. They are effective in the treatment of infectious diseases while simultaneously mitigating many of the side effects that are often associated with synthetic antimicrobials. The plant systematic classification is as follows. Kingdom—Plantae (Plants), Subkingdom—Tracheobionta (vascular plants), Division—Spermatophyte (seed plant), Subdivision—Angiosperm, Class—Dicotyledoneae, Order—Caryophyllales, Family—Portulacaceae, Genus—*Portulaca*, Species—*oleracea*. The plant* P. oleracea L.* (Purslane) is commonly known as Porsulane, an herbaceous weed. This plant is an annual succulent prostrate herb; stem is about 15.30 cm long, reddish, swollen at the nodes, and quite glabrous. Leaves are fresh, subsessile, 6.25 mm long, and alternate or subopposite. Flowers are sessile, axillary and terminal, few-flowered heads. Microscopic analysis of the leaf powder invariably shows spherical mineral crystals, sieve plants, tracheas with spiral, annular, and scalar form thickening, and vessels with bordered pits [10]. The present study deals with the isolation, purification and identification of flavonoid apigenin from* P. oleracea L*. (Figure 6) and also determines antibacterial activity on pathogenic bacteria.

## 2. Materials and Methods

### 2.1. Plant Material

Healthy aerial part of the plant of* P. oleracea L.* was collected from around Gulbarga University campus during the month of June 2012. The plant material was identified and authenticated from the Department of Botany, Gulbarga University, Gulbarga, Karnataka (India); voucher specimen (number HGUG-5013) has been deposited in herbarium of the same department.

### 2.2. Chemicals

Methanol, ethanol, ethyl acetate, petroleum ether, diethyl ether, H_2_SO_4_, chloroform, HCl, KOH, hexane, silica gel 60–120 mesh, tween 80, phosphate buffer saline, FCR reagent, all the chemicals, solvents, and reagents were analytical grade and were obtained from Hi media.

### 2.3. Isolation of Total Flavonoids by Soxhlet Extraction Method

Before extraction,* P. oleracea L.* was crushed into powder by versatile plant pulverize. The powder of the sample was degreased by soxhlet extractor with petroleum ether until the color of elute becomes colorless. The same powder sample was accurately weighed and placed in soxhlet extractor by adding 80 mL of ethanol: water (70 : 30) solvent, followed by the extraction for up to 5 h, and then extract solution was concentrated. The extract was centrifuged at 11000 rpm for 30 min; supernatant was taken for further use [11].

### 2.4. Qualitative Test for Flavonoids

Two methods were used to determine the presence of flavonoids in the extract; 5 mL of dilute ammonia solution was added to a portion of total flavonoid extracts followed by addition of concentrated H_2_SO_4_ [12, 13].

### 2.5. Determination of Total Flavonoid Content by UV-Spectrophotometric Method

Firstly, 2 mL of the sample solution was accurately removed in a volumetric flask (10 mL) by adding 0.6 mL of NaNO_2_ (5%) solution, shaking up, and then standing for 6 min. Secondly, 0.5 mL of the Al(NO_3_)_3_ (10%) solution was added to the volumetric flask, shaken, and left to stand for 6 min. Finally, 3.0 mL of the NaOH (4.3%) solution was added to the volumetric flask, followed by addition of water to the scale, shaken, and left to stand for 15 min before determination. Using the sample solution without coloration as reference solution and 500 nm as determination wavelength, the coloration method was used to determine the content of flavonoids in the sample by ultraviolet-visible detector [11].

### 2.6. Separation of Bioactive Compounds by PTLC

Glass plates (20 × 20 cm) thickly coated (0.4–0.5 nm) with silica gel “G” (45 g/80 mL water) and activated at 100°C for 30 minutes and cooled at room temperature were used for preparative thin layer chromatography (PTLC). The extract of flavonoid and standard apigenin were applied on plate and developed in n-butanol-water-acetic acid solvent system (mobile phase) 12 : 2 : 1 v/v/v. The chromatogram was air-dried and visualized under visible and UV light and also in iodine chamber. The spots were marked and the *R*_*f*_ values were calculated [14].

### 2.7. Separation of Flavonoids by Column Chromatography

The total flavonoids which are isolated can be purified and separated by column chromatography separation method. The 45 cm length and 3 cm width of the column were used and it is filled with the slurry of silica gel-H of mesh size 60–120 *μ* (Hi media, Mumbai) to 1/3 portion using n-hexane. Care should be taken to avoid the air bubble formation during column packing. Set the column by the solvent n-hexane. 10 g of total flavonoid extract was bound with silica gel and loaded on the top of the column. The column was eluted with gradient solvent system of n-butanol-water-acetic acid system 12 : 2 : 1 v/v/v until the color of the column is colorless.

### 2.8. Physicochemical Structure Elucidation of Flavonoids by Spectral Analysis

The pure compound of* P. oleracea L.* isolated was subjected to IR, NMR, LC-MS, and HPLC studies to obtain spectral data for the detection of functional group, number of protons, molecular mass of the compound, and purity, respectively [15].

### 2.9. Antimicrobial Susceptibility Test

#### 2.9.1. Microorganisms

The bacterial strains employed in the current study were procured from Institute of Microbial Technology (IMTECH), Chandigarh (India), which include* Pseudomonas aeruginosa *(MTCC 424),* Klebsiella pneumoniae *(MTCC109),* Salmonella typhimurium *(MTCC98),* Proteus mirabilis *(MTCC425), and* Enterobacter aerogenes *(MTCC111). These species were originally isolated from clinical samples and identified by standard biochemical reactions.

#### 2.9.2. Media

Nutrient broth (Hi Media M002) contains peptic digest of animal tissue (5 g/L); yeast extract (1.50 g/L) and beef extract (1.5 g/L) were used for the growth of bacterial cultures. Antibiotic assay media No. 11 (Hi Media MM004) containing Peptic digest of Animal tissue (6 g/L), Casein enzyme hydrolysed (4 g/L), Yeast extract (1.50 g/L), Dextrose (1.00 g/L). Agar (15.00 g/L) was used for antibacterial activity.

#### 2.9.3. Disc Diffusion Assay

The antimicrobial activity of apigenin was evaluated using a slightly modified agar disc diffusion method [16]. A bacterial culture grown for 18 h was serially diluted in 9 mL of 0.1% peptone to obtain 10^5^ CFU/mL and 100 *μ*L spread on the surface of Mueller Hinton (MH) agar in Petri plates. An aliquot (10 *μ*L) of apigenin was pipetted on a sterile paper disc (Whatman No. 1, 5.5 mm paper disc) on the agar surface. A disc impregnated with an aliquot (10 *μ*L) of streptomycin (Fluka, Switzerland) served as a positive control on the same plate. The plates were inverted and incubated for 18 h at 37°C. Microbial inhibition was determined by measuring the diameter of the clear zone of inhibition of growth around each disc and recorded as diameter of inhibition zone (DIZ) in millimeter. All assays were performed using a randomized complete block design with two replicates.

#### 2.9.4. Broth Dilution Assay

To determine the minimal inhibitory concentration (MIC), quantitative serial dilutions of apigenin were tested against five pathogenic bacterial strains with some modifications of the method described by Hufford and Clark [17, 18]. Twofold serial dilution of apigenin was made with MH broth. After adding 20 *μ*L of apigenin to the first tube containing 1 mL of MH broth, serial transfers were made through to the fourth tube. A 0.5 mL aliquot (5 × 10^5^ CFU/mL) of test microorganism was added to each tube. A control tube contained 10 *μ*L of streptomycin in MH broth and microorganism was maintained. Tubes were subsequently incubated at 37°C. The tubes were visually examined for the lowest concentration of apigenin that showed inhibition of microbial growth (indicated by a clear solution) after 24 h and 48 h. The concentration in the lowest serial dilution of the apigenin at which growth did not occur on broth was recorded as the MIC. Finally, the pH value of the inoculated broth containing the MIC of apigenin was measured. Buffering of broth containing apigenin of* P. oleracea L.* was done using NaOH and subjected to broth dilution assay as the above to determine the MIC values.

## 3. Results

### 3.1. Qualitative Test for Flavonoids

The yellow coloration is observed after adding H_2_SO_4_ and it disappeared on standing. Few drops of 1% aluminum solution were added to a portion of flavonoid extract and again yellow coloration was observed; this indicates the presence of flavonoids.

### 3.2. Separation of Bioactive Compound by PTLC

When the developed plates were sprayed with 5% ethanol ferric chloride solution and placed in iodine chamber, they showed a spot which has a brownish color. The *R*_*f*_ value (0.82) of apigenin isolated from the* P. oleracea L.* coincided with the *R*_*f*_ value of standard apigenin. The results of PTLC *R*_*f*_ values are shown in Table 1.

### 3.3. Determination of Total Flavonoid Content by UV-Spectrophotometric Method

**Figure 1:**
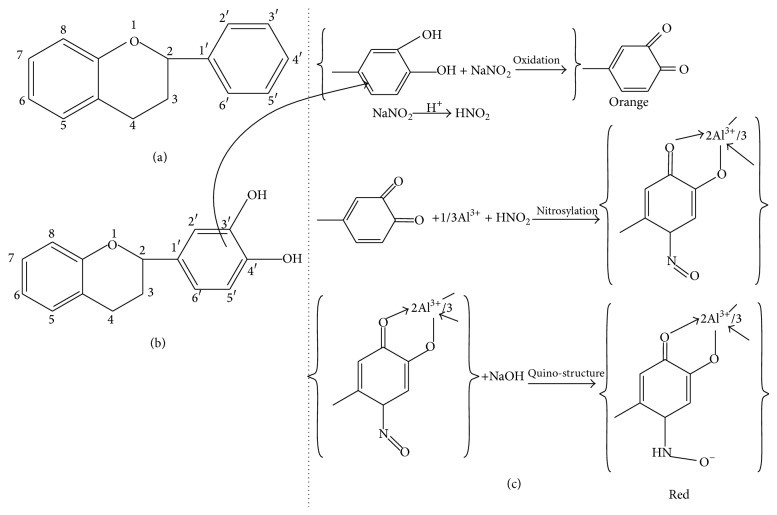
The color reaction of flavonoids and chromogenic system.

The basic structure of flavonoids was presented in Figure 1(a), and most of the flavonoids present in* P. oleracea L.* will be having 3′,4′-dihydroxy-substituted structure as shown in Figure 1(b). Flavonoids with 3′,4′-dihydroxy-substituted structure have shown a special color by reacting with the system of NaNO_2_-Al(NO_3_)_3_-NaOH. The color reaction of flavonoids and chromogenic system is presented in Figure 1(c). This method is based on the reaction of aluminum ion with flavonoid at alkaline medium forming red chelates. By measuring the absorption of such red chelates, it is possible to determine the flavonoids.

### 3.4. Separation of Flavonoids by Column Chromatography

The total flavonoids of ethanol extract of* P. oleracea L.* of about 20 g were fractionated on a silica gel-H (60–120 mesh) column at room temperature and pressure 260C, 1 bar. The total 32 fractions of 100 mL of each were collected. Each of the elutes was then crystallized with chloroform. The purified compound was subjected to its spectral analysis for structural elucidation.

### 3.5. Physicochemical Structure Elucidation of Flavonoids by Spectral Analysis

**Figure 2:**
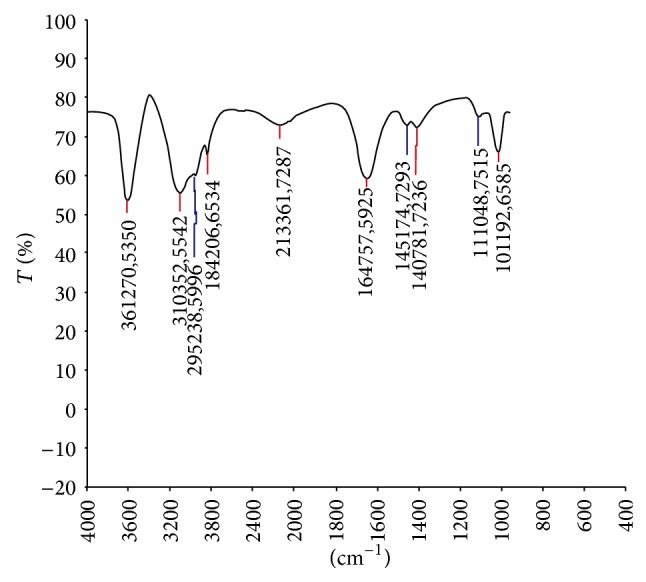
FT-IR spectrum of isolated compound apigenin from* P. oleracea L*.

*FT-IR*. It can be resolved from the IR spectra that there are intensive bands in the 110–1000 cm^−1^ span that are characteristics of glycoside bonds and not bands of any sugar type. There are bonds of V_CH_ vibration of CH_2_ group at approximately 2952 cm^−1^ and 2842 cm^−1^ and *δ*_CH_ vibration at approximately 1451 cm^−1^. In the spectrum of V_OH_ vibration, there are bonds which are found at approximately 3612 cm^−1^ and others at approximately 3103 cm^−1^ that are most probably the result of V_OH_ vibration of phenol OH groups. The intensive band at approximately 1647 cm^−1^ is most probably the result of V_C=O_ vibration of C=O group from central heterocyclic ring, while the V_C−O_ vibration occurs at approximately 1110 cm^−1^. With these above spectral characteristics, it is indicated that the probable compound is apigenin (Figure 2).

**Figure 3:**
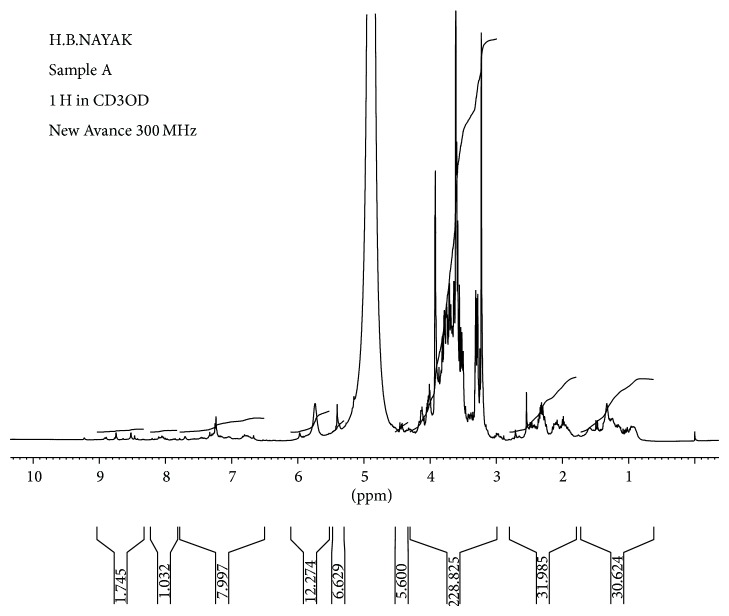
NMR spectrum of isolated compound apigenin from* P. oleracea L.*

^*1*^ *H NMR*. The ^1^H NMR spectrum shows two singlets at 1.032 and 1.745 *δ* (ppm) resonated to multiple of two protons of aromatic systems. The compound singlet peak at *δ* 7.99 ppm (^*1*^ *H*) was indicated for aromatic proton of phenolic hydroxyl. The identification of purified compound was further confirmed by ^1^H spectrum data available in literature (Figure 3).

**Figure 4:**
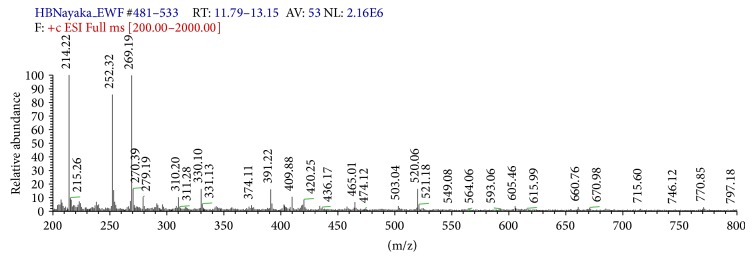
LC-MS spectrum of isolated compound apigenin from* P. oleracea L.*

*LC-MS*. The mass spectrum that displayed a molecular ion (M^+^) peak at* m/z* 269.19 indicates the molecular weight of the compound corresponds to the molecular weight of apigenin and also corresponds to molecular formula C_15_H_10_O_5_. This further confirms that the structure of the isolated compound is apigenin (Figure 4).

**Figure 5:**
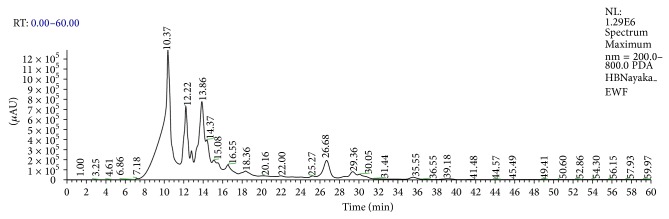
HPLC chromatogram of isolated compound apigenin from* P. oleracea L.*

**Figure 6:**
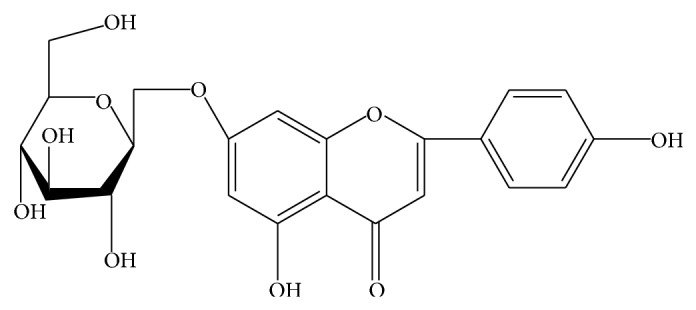
Structure of flavonoid apigenin isolated from* P. oleracea L.*

*HPLC*. Chromatographic system and conditions are the LC system consisted of a Waters 600 solvent delivery pump, a Waters 2487 UV-visible spectrophotometric detector, an Empower system controller (Waters, USA), and HPLC analytical column (Diamonsil C 18 Column, 4.6 × 200 mm, i.d., 5 *μ*M particle size, Dikma) connected with a guard column filled with the same chromatographic stationary phase. The mobile phase for HPLC analysis consisting of Methanol-0.2% Phosphoric acid (45  : 55, v/v) were filtered under reduced pressure and degassed by ultrasonic before use. HPLC analysis with UV detection at 350 nm was performed at a flow rate of 1.0 mL/min. HPLC column temperature was 35°C. The sample injection volume was 10 *μ*L. The chromatographic peak of the apigenin is shown in the chromatogram in Figure 5.

### 3.6. Antibacterial Susceptibility Test

#### 3.6.1. Disc Diffusion Assay

The mean diameters of the inhibition zones of apigenin against five pathogenic bacterial strains are shown in Table 2. The results from disc diffusion assay showed that, among all bacterial strains,* S. typhimurium *(17.36 ± 0.18) and* P. mirabilis *(19.12 ± 0.01) have shown significant (*P* < 0.05) zone of inhibition, where remaining bacterial strains have shown less significant (*P* < 0.05) zone of inhibition when compared with control values (14.56 ± 0.21) and (11.68 ± 0.13), respectively.

#### 3.6.2. Broth Dilution Assay

The results of the MIC values of apigenin of* P. oleracea L.* obtained at 24 h and 48 h are presented in Table 3. The present experimental data demonstrated that the apigenin of* P. oleracea L.* has displayed the antibacterial activity with MIC value > 4 mg/mL against all pathogenic bacterial strains which were subjected in the present study. The MIC values did not change after 48 h and also did not correlate well with the diameter of inhibition zones from the disc diffusion assay.

## 4. Discussion

The present study is conducted to extract flavonoids of* P. oleracea L.* by modified method of previous study [11]. This method of extraction was the best which had the highest content of quercetin, rutin, and apigenin, which are glycol-flavones with a low impurity. More than 2000 flavonoids have been reported among woody and nonwoody plants [19]. PTLC, UV, and IR spectral studies have provided a new dimension to the chemistry of flavonoids to such an extent that their presence has become important in taxonomic study [20]. Presence of flavonoids has been reported in many plant species* like Lycium barbarum, Passiflora palmer, Cassia angustifolia, *and* Jatropha curcas L*. [21–24]. Apigenin has been reported in plant species like* Bellis perennis L.* and* Adinandra nitida *[15, 25]. It is well known that apigenin has an antioxidant, anticarcinogenic, and spasmolytic activities and can reduce high blood pressure. That is why leaves of * P. oleracea L. are* a good source of apigenin, which can be added to food as a kind of functional ingredient or used as a vegetable, which have many beneficial effects on human health. It can also be used in medicine in standard forms of administration, such as capsules, tablets, and oral suspensions [26]. The extraction, isolation, purification, and characterization of this compound from* P. oleracea L.* and structural elucidation of this compound have been studied. In the present study, the results of qualitative tests (specific for flavonoids) of flavonoids can be compared with the results of previous studies conducted on phytotoxicity and antimicrobial activities of flavonoids in* Ocimum gratissimum* [27]. In the present study, the flavonoids contents are determined by NaNO_2_-Al(NO_3_)_3_-NaOH colorimetric method. The result indicates that the* P. oleracea L.* has contained more flavonoids. Basic structure of flavonoids was presented in Figure 1(a), and most of the flavonoids in* P. oleracea L*. have 3′,4′-dihydroxy-substituted structure as shown in Figure 1(b). Flavonoids with 3′,4′-dihydroxy-substituted structure can show special color by reacting with the system of NaNO_2_-Al(NO_3_)_3_-NaOH. The color reaction of flavonoids and chromogenic system is presented in Figure 1. As shown in Figure 1, this method is based on the reaction of aluminum ion with flavonoid at alkaline medium forming red chelates. By measuring the absorption of such red chelates, it is possible to determine the flavonoids [11]. The structural elucidation of apigenin is possible because of spectral data obtained. In the present study the IR spectrum has vibration bonds at 3612 cm^−1^ and 3103 cm^−1^ approximate, which are most probably the result of V_OH_ vibration of phenol OH groups. The intensive band at approximately 1647 cm^−1^ is most probably the result of V_C=O_ vibration of C=O group from central heterocyclic ring. Similar results were obtained in the study conducted for extraction of apigenin from the sage*(Salvia officinalis L.)* from Jordan [14]. The LC-MS spectrum that displayed the molecular ion (M^+^) peak at* m/z* 269.19 in the present study indicates the molecular weight of the apigenin compound; this result is similar to the LC-MS spectrum study conducted by [15] in the plant* Adinandra nitida.* For the confirmation of purity, the compound is again subjected to HPLC analysis; the results of HPLC chromatogram showed single base peak at 10.3 AU; it is again conformed with the compound apigenin. Nowadays, multiple drug resistance developed due to the indiscriminate use of drugs commonly used in the treatment of infectious diseases. Unfortunately, bacteria have genetic ability to transmit and acquire resistance to drugs and chemicals [28]. Based on the results of antibacterial studies shown in Table 2, the apigenin had an antibacterial activity more significant on* S. typhimurium *and* P. mirabilis* when compared with control group. This may be due to the chemical nature of apigenin, cell membrane permeability, and other factors. In general, composition of the inhibition zones diameter showed that apigenin was more effective against both gram positive and gram negative bacteria. This difference may be due to several possible reasons such as permeability barrier provided by the presence of cell wall with multilayer structure in gram negative bacteria or the membrane accumulation mechanism or presence of enzymes in periplasmic space which are able to break down foreign molecules introduced from outside [29]. The MIC value (Table 3) for apigenin isolated from* P. oleracea L.* against all the pathogenic bacterial strains which were subjected in the present study was >4 mg/mL; it is reported that, for bacterial antimicrobials, the MIC was often near or Aquila values [30].

## 5. Conclusion

The flavonoids of* P. oleracea L.* were isolated by employing the extraction with ethanol : water (70 : 30) solution. The result of qualitative test which is specific for flavonoids confirmed that the isolated compound was flavonoid. The results of PTLC, HPLC, LC-MS, FT-IR, and NMR further confirmed that the compound is apigenin. The antibacterial results of the present study will suggest that the isolated apigenin bioactive compound is also having potential antibacterial activity indicating that it can be used for development of antibacterial drugs for the treatment of diseases associated with these pathogenic bacteria.

## Figures and Tables

**Table 1:** *R*_*f*_ values of isolated bioactive compound and standard apigenin.

Sl. number	Samples	Solvent system (12 : 2 : 1 v/v/v)	*R*_*f*_ values
1	Standard apigenin	n-butanol-water-acetic acid	0.82
2	Isolated compound	n-butanol-water-acetic acid	0.83

**Table 2:** Antimicrobial activity (diameter of inhibition zone) of apigenin of *P. oleracea L.* on pathogenic bacteria.

Bacterial strains	MTCC number	Diameter of inhibition zone (mm)	
		Streptomycin as control (10 µL)	Apigenin (10 µL)
*P. aeruginosa *	MTCC424	17.42 ± 0.54	12.24 ± 0.41
*K. pneumoniae *	MTCC109	21.33 ± 0.33	10.52 ± 0.38
*S. typhimurium *	MTCC98	14.56 ± 0.21	17.36 ± 0.18^**^
*P. mirabilis *	MTCC425	11.68 ± 0.13	19.12 ± 0.01^***^
*E. aerogenes *	MTCC111	19.66 ± 0.27	14.02 ± 0.03

^**^Moderate significant; ^***^most significant at *P* ≤ 0.05. Values are expressed as mean ± SD.

**Table 3:** Antibacterial activity (minimum inhibition concentration after 24 h and 48 h; mg/mL) of apigenin of *P. oleracea L.*

Bacterial strains	MTCC number	Apigenin of *P. oleracea* L.
*P. aeruginosa *	MTCC424	>4
*K. pneumonia *	MTCC109	>4
*S. typhimurium *	MTCC98	>4
*P. mirabilis *	MTCC425	>4
*E. aerogenes *	MTCC111	>4
